# Optimizing antidepressant use in geriatric depression: expert consensus from French societies of geriatrics, old age psychiatry, and clinical pharmacy (SFGG, SF3PA, and SFPC)

**DOI:** 10.1192/j.eurpsy.2026.12206

**Published:** 2026-03-26

**Authors:** Morgane Houix, Alexis Lepetit, Christophe Arbus, Sophie Armand-Branger, Sylvie Bonin-Guillaume, Benjamin Calvet, Mathieu Corvaisier, Ismael Conejero, Anne-Laure Debruyne, Sandrine Louchart de la Chapelle, Gérald Deschietere, Thomas Desmidt, Jean-Michel Dorey, Olivier Drunat, Jean-Pascal Fournier, Quentin Gallet, Jacques Gauillard, Cécile Hanon, Nicolas Hoertel, Ilia Humbert, Isabelle Jalenques, Aline Lepelletier, Frédéric Limosin, Nicolas Marie, Stéphanie Miot, Jean Olivier, Laurence Petit, Beatriz Pozuelo Moyano, Maximilien Redon, Gabriel Robert, Jean Roche, Tanguy Taillefer de Laportaliere, Pierre Vandel, Sonia Prot-Labarthe, Samuel Bulteau

**Affiliations:** 1 Pharmacy, CHU Nantes: Centre Hospitalier Universitaire de Nantes, France; 2 INSERM UMR 1246 SPHERE Methods in Patient-centered Outcomes and Health Research, Nantes, France; 3 Hospices Civils de Lyon, France; 4 Centre Hospitalier Universitaire de Toulouse Pôle Psychiatrie: Centre Hospitalie, France; 5 Pharmacie, Mental Health Center, Sainte-Gemmes sur Loire, France; 6 AP-HM: Assistance Publique Hopitaux de Marseille, France; 7 CH Esquirol: Centre Hospitalier Esquirol, France; 8 CHU Angers: Centre Hospitalier Universitaire d’Angers, France; 9 Centre Hospitalier Universitaire de Nimes, France; 10 CH Charles Perrens: Centre Hospitalier Charles Perrens, France; 11 Princess Grace Hospital Centre: Centre Hospitalier Princesse Grace, Monaco; 12 Brussels Saint-Luc University Hospital: Cliniques Universitaires Saint-Luc, Belgium; 13 CHU Tours: Centre Hospitalier Regional Universitaire de Tours, France; 14 CH Vinatier: Centre Hospitalier Le Vinatier, France; 15 Hopital Bretonneau, France; 16 Département de Médecine Générale, Faculté de Médecine, Nantes University: Nantes Universite, France; 17 Université d’Angers, POPS, SFR ICAT, Angers, France; 18 CHU Angers: Centre Hospitalier Universitaire d’Angers, France; 19 Hopitaux Universitaires Est Parisien, France; 20 Corentin Celton Hospital Adults and Elderly Subjects Psychiatry Service: Hopital, France; 21 Hôpitaux Universitaires de Strasbourg: Les Hopitaux Universitaires de Strasbourg, France; 22 CHU Clermont-Ferrand: Centre Hospitalier Universitaire de Clermont-Ferrand, France; 23 CHU Nantes: Centre Hospitalier Universitaire de Nantes, France; 24 CH Guillaume Regnier: Centre Hospitalier Guillaume Regnier, France; 25 CHU Montpellier: Centre Hospitalier Universitaire de Montpellier, France; 26 CHU Toulouse: Centre Hospitalier Universitaire de Toulouse, France; 27 EPS Roger Prévot, Mental Health Center, Moisselles, France; 28 Lausanne University Hospital, Department of Psychiatry: Centre Hospitalier Univer, Switzerland; 29 CHU de Lille Hôpital Fontan 1 et 2: Centre Hospitalier Universitaire de Lille Ho, France; 30 CHUV: Centre Hospitalier Universitaire Vaudois, Switzerland

**Keywords:** guidelines, late-life depression, old-age psychiatry

## Abstract

**Background:**

Late-life depression refers to a depression occurring in older adults, defined as individuals aged 65 years and older. It is common and often mismanaged due to complex clinical profiles, polypharmacy, and limited evidence-based guidance. Existing tools poorly address psychiatric nuances in older adults. This work aims to develop French recommendations based on experts’ consensus on the use of antidepressants in unipolar late-life depression, to guide safer prescribing practices.

**Methods:**

This tool was developed using a Delphi survey, based on a review of literature published between 2014 and 2024 and focused on “antidepressants” and “late-life depression” Experts from various fields: geriatric psychiatry, clinical pharmacy and general practice, rated items using a 9-point Likert scale. Items with a median score ≥ 7 and at least 80% agreement were validated and included in the final version of the tool.

**Results:**

Twenty to 23 experts per round, different from the authors of the proposed items, participated in a four-round Delphi process. The resulting tool includes 57 validated items across 10 sections and a stepped-care algorithm for treating major depression in older adults. It addresses drug choice, dosing, monitoring, comorbidities, and treatment resistance, prioritizing safe first-line options like sertraline, citalopram, and escitalopram.

**Conclusion:**

A Delphi survey involving multidisciplinary experts led to a French consensus tool for prescribing antidepressants in unipolar late-life depression. It integrates clinical evidence and expert judgment to address treatment complexity, drug safety, and resistance. The tool offers practical, stepwise recommendations tailored to primary care, aiming to optimize antidepressant use, reduce iatrogenesis, and improve patient outcomes.

## Keypoints for clinicians

- Start with SSRIs (particularly sertraline, citalopram, or escitalopram) as first-line treatment due to their favorable safety profile.
- Begin at half the adult dose and increase gradually, but aim to reach therapeutic doses within 4–12 weeks.
- Monitor for hyponatremia when using SSRIs in this population, particularly with concomitant diuretics.
- Consider augmentation strategies rather than switching when a partial response is achieved.
- Regular follow-up (every 1–3 months) is crucial for monitoring efficacy and adverse effects.
- For treatment-resistant depression, consider lithium augmentation or atypical antipsychotics after failed trials with two antidepressants.
- ECT remains the gold standard for severe or psychotic depression in older adults.

## Introduction

Late-life depression (LLD) is a prevalent public health concern affecting a substantial proportion of adults aged 65 and older [1]. Estimates of major depression in individuals aged 75 years and older range from 4.6 to 9.3% [2]. While antidepressants remain the most extensively studied therapeutic option, other interventions such as psychotherapy, exercise therapy, and electroconvulsive therapy have also demonstrated efficacy. Psychotherapy is generally recommended for mild to moderate depression. However, in cases of severe depression, antidepressant treatment is often required, given the association between persistent depressive symptoms and increased mortality, functional decline, caregiver burden, and the exacerbation of comorbid medical conditions. [3]. In France, depression management relies on coordinated care, with general practitioners leading screening, diagnosis, and follow-up, and psychiatrists involved in cases of severe mental illness or resistance to a first-line treatment. Geriatric psychiatrists have the specific goal to assist community psychiatrists and general practitioners in cases of complexity induced by aging and polypathologies. However, training of community psychiatrists and general practitioners in old age psychiatry, and access to quick specialized advice is very challenging given the lack of geriatric psychiatry professionals and facilities. In France, there are significant barriers to collaboration between primary care providers and specialists [4]. Given the population aging, GPs who see a high volume of patients with multiple comorbidities require decision-making guidance (regarding drug selection, dosage, based on common and pragmatic scenarios). Psychologists provide psychotherapeutic support, while other healthcare professionals, including pharmacists and nurses, contribute to monitoring, patient education, and overall care. Although clinical pharmacy is not yet widely established in France, clinical pharmacists can play a key role in medication reviews and the optimization of prescribing, as demonstrated in the UK and in community–hospital care coordination. The European Association of Clinical Pharmacy has recently advocated for the broader implementation of clinical pharmacy in mental health and for the development of supporting tools for pharmacists [5].

As in the general population, where patients receive adequate treatment in only 40% of cases [6], depression in older adults is frequently underdiagnosed and undertreated due to atypical symptom presentation, comorbidities, and the misconception that depressive symptoms are a normal part of aging [7]. Poor management reduces quality of life and increases morbidity and cognitive decline [8, 9]. Treatment is further complicated by polypharmacy, age-related physiological changes, and the need to consider drug interactions and contraindications [10]. Additionally, older adults with polypharmacy are underrepresented in clinical trials, limiting the applicability of adult guidelines – a concern relevant to depression as well as other psychiatric conditions [11, 12].

To support prescribing in older adults, tools such as the Laroche criteria (updated in 2023 with REMEDI[e]S [13]), STOPP/START [14], and internationally the Beers criteria [15], are commonly used. Psychotropic medications are a major focus due to their high iatrogenic risk [16–20]. A 2019 regional study of 347 older psychiatric inpatients found that over 70% had at least one potentially inappropriate psychotropic prescription (PIP Independent review of a subset of records determined 60% were clinically appropriate, 32.4% partially relevant, and 8.8% unjustified [21], highlighting that PIP detection tools alone are insufficient in old-age psychiatry, where treatment resistance, severe behavioral disorders and complications, and monitoring must also be considered.

Following the example of other European countries, such as Germany, with the Priscus list [22] and Italy [23], the development of a national tool, using a Delphi study, for routine clinical use was deemed necessary. Given the high burden of disability and mortality associated with major depression in older adults, lack of formation, lack of geriatric psychiatrists and specialized pharmacists, suboptimal prescribing is a serious public health concern. This French-speaking Delphi study therefore aimed to address common questions that general practitioners, geriatricians, and community psychiatrists can face in daily practice with older adults suffering from unipolar depression, to facilitate pharmacological management in primary care.

## Materials and methods

The Delphi approach was chosen to obtain expert consensus [24]. The survey focused on the prescription of antidepressants in older adults over the age of 75 or those over 65 with multiple pathologies (i.e., suffering from at least three chronic conditions).

### Development of the items

The initial research team (MH, SPL, SB) performed a literature search on PubMed, covering articles published from 2014 to January 2024, and based on the main known risks associated with underuse and misuse of antidepressants in this population, taking into account the main comorbidities faced in this population. The keywords “antidepressants” and “late-life depression” were used, along with additional terms related to specific conditions such as “hyponatremia,” “renal disease,” “hepatic impairment,” “bleeding” and “cardiovascular effects.” This comprehensive approach ensured coverage of both general treatment guidelines and specific safety considerations relevant to older populations. The selected articles were mainly systematic literature reviews based on previously published meta-analyses and guidelines (*n* = 9) [3, 25–32], 1 open clinical trial [33], 2 health and reimbursement data studies [34, 35], 4 prescription guides [36–39], and 20 more specific articles on clinical situations [40–59].

The initial items developed were submitted for validation by a small group of four voluntary experts in old age psychiatry to ensure clarity and relevance in item formulation. After this review, an initial questionnaire of 53 items was developed. The items were divided into the following sections: framework for antidepressant prescription (6 items), prescription modalities for antidepressants (15 items), prescription in case of cardiovascular history (8 items), in case of altered neurocognitive assessment (2 items), in case of renal insufficiency (4 items), in case of hepatic insufficiency (2 items), in case of co-prescriptions of interest (6 items), in case of other comorbidities of interest (1 item), in case of failure of two lines of monotherapies (4 items), in case of partial efficacy or failure of antidepressant combination therapy (5 items). We have also chosen to provide a decision tree that brings together some of the information contained in the items in a more readable format.

### Selection of the expert group

Several types of experts were sought to ensure comprehensive input: psychiatrists specialized in old age psychiatry members of the French-speaking Society of Psychogeriatrics and old age Psychiatry (SF3PA), pharmacists working in psychiatry or geriatrics members of the French Society of Clinical Pharmacy (SFPC), geriatricians members of SF3PA or the French Society of Geriatrics and Gerontology (SFGG), or general practitioners with experience working with SFPC member pharmacists. All had recognized expertise in this field given their specific professional experience, publications, and/or position in a university hospital and/or in specialized clinical research units. The predominance of geriatric psychiatrists (52.2–61.9% across rounds) reflects their specialized expertise in this field, while we actively sought to include other disciplines to ensure multidisciplinary perspectives. The goal was to obtain a geographically diverse sample to ensure the representativeness of practices and the generalizability of recommendations. A minimum of 20 experts per round was expected.

### Data collection

Four rounds of consultations took place between March 15 and October 29, 2024. A delay of 15–30 days was allowed for each consultation round. The items and their scientific rationale were sent to the experts, and responses were collected online via Google Form®. Experts were encouraged to provide open-ended suggestions in each round to ensure all participants could express their views if necessary, enriching the qualitative aspects of the consensus process.

In each round of consultation, the following data were collected for each expert: name, specialty, number of years of experience, city of practice, type of practice, and conflicts of interest.

Experts indicated their level of agreement with the proposed items. A score reflecting this agreement was assigned by the experts for each item on a Likert scale from 1 to 9: 1 if the expert strongly disagrees with the item, 10 if they strongly agree with the item. If the response score was lower than 7/9, a justification from the expert was requested in the form of free text.

### Procedure

For each item, we calculated the median scores and the level of agreement among experts, defined as the proportion of experts giving a score equal to or greater than 7/9. To reach consensus, an item required a median score of at least 7 out of 9 and agreement from at least 80% of the experts. In each consultation round, items that did not reach consensus were modified according to the experts’ feedback and submitted for a new consultation round. The initial research team, who did not participate in the survey, analyzed the responses of experts and modified item. If less than 30% of experts gave a score higher than 7/9 and the comments did not provide a relevant modification for the item, the item was removed. This iterative process ensured that only items with strong expert support were included in the final tool.

### Regulatory considerations

An expert opinion was sought from the local Ethics, Deontology, and Scientific Integrity Committee. Since this survey was exempted under the Jardé law (Art. L1121 of the French Public Health Code), no approval from the Committee for the Protection of Persons or the National Commission on Informatics and Liberty was required. The main regulatory steps to ensure were therefore related to the experts involved in the Delphi method: their absence of conflicts of interest was verified and their consent was obtained. The survey questionnaire began with a legal notice about the management of data obtained through the survey. Answers and intermediate results were presented in an anonymized form to all participants.

## Results

### Participating experts

A total of 20–23 experts participated in the four rounds of consultations. This participation rate remained stable throughout the process, indicating important engagement and commitment to the consensus development. The majority of experts (61.9–75.0% across rounds) had more than 10 years of experience in their respective fields. Table 1 describes the profile of the participants.

**Table 1.** Characteristics of the expert panel Table 1. long description.

	1st round*n* = 23	2nd round*n* = 20	3rd round*n* = 21	4th round*n* = 21
Specialty, *n* (%)				
Geriatric psychiatrist	12 (52.2)	11 (55.0)	13 (61.9)	13 (61.9)
Hospital pharmacist	6 (26.1)	5 (25.0)	5 (23.8)	5 (23.8)
Geriatrician	3 (13.0)	3 (15.0)	2 (9.5)	3 (14.3)
General practitioner	2 (8.7)	1 (5.0)	1 (4.8)	–
Years of experience, *n* (%)				
< 5 years	6 (26.1)	5 (25.0)	4 (19.0)	6 (28.6)
(5–10) years	1 (4.3)	–	4 (19.0)	1 (4.8)
> 10 years	16 (69.6)	15 (75.0)	13 (61.9)	14 (66.7)
Type of practice, *n* (%)				
Hospital	21 (91.3)	19 (95.0)	20 (95.2)	21 (100)
Primary care practice	2 (8.7)	1 (5.0)	1 (4.8)	–
French region or country (city) of practice, *n* (%)				
Auvergne-Rhônes-Alpes (Lyon)	1 (4.3)	1 (5.0)	2 (9.5)	1 (4.8)
Bretagne (Rennes)	1 (4.3)	1 (5.0)	1 (4.8)	1 (1.8)
Centre-Val de Loire (Tours)	–	1 (5.0)	1 (4.8)	1 (4.8)
Grand Est (Strasbourg)	1 (4.3)	1 (5.0)	1 (4.8)	1 (1.8)
Hauts de France (Lille)	–	–	–	1 (4.8)
Ile-de-France (Paris and suburbs)	5 (21,8)	3 (15.0)	3 (14.3)	3 (14.3)
Nouvelle Aquitaine (Bordeaux, Limoges)	2 (8.7)	2 (10.0)	2 (9.5)	2 (9.5)
Occitanie (Toulouse, Nîmes, Montpellier)	5 (21.8)	4 (20.0)	4 (19.0)	4 (19.0)
Pays de la Loire (Angers, Nantes)	5 (21.8)	3 (15.0)	4 (19.0)	3 (14.3)
Provence Alpes Côte d’Azur (Marseille)	1 (4.3)	1 (5.0)	1 (4.8)	1 (4.8)
Belgium (Bruxelles)	–	–	–	1 (4.8)
Monaco	1 (4.3)	1 (5.0)	1 (4.8)	1 (4.8)
Swiss (Lausanne)	1 (4.3)	2 (10.0)	1 (4.8)	1 (4.8)

#### Validated recommendations

**Figure 1.**
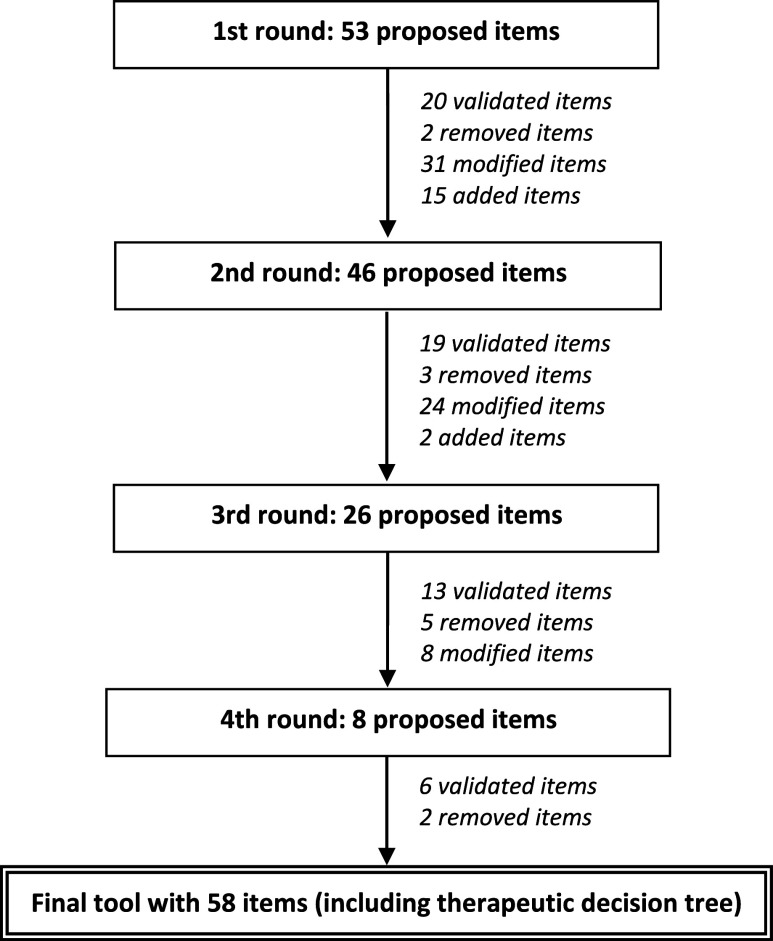
Flowchart of the Delphi consensus process. Figure 1. long description.

Figure 1 presents the entire Delphi process.

**Figure 2.**
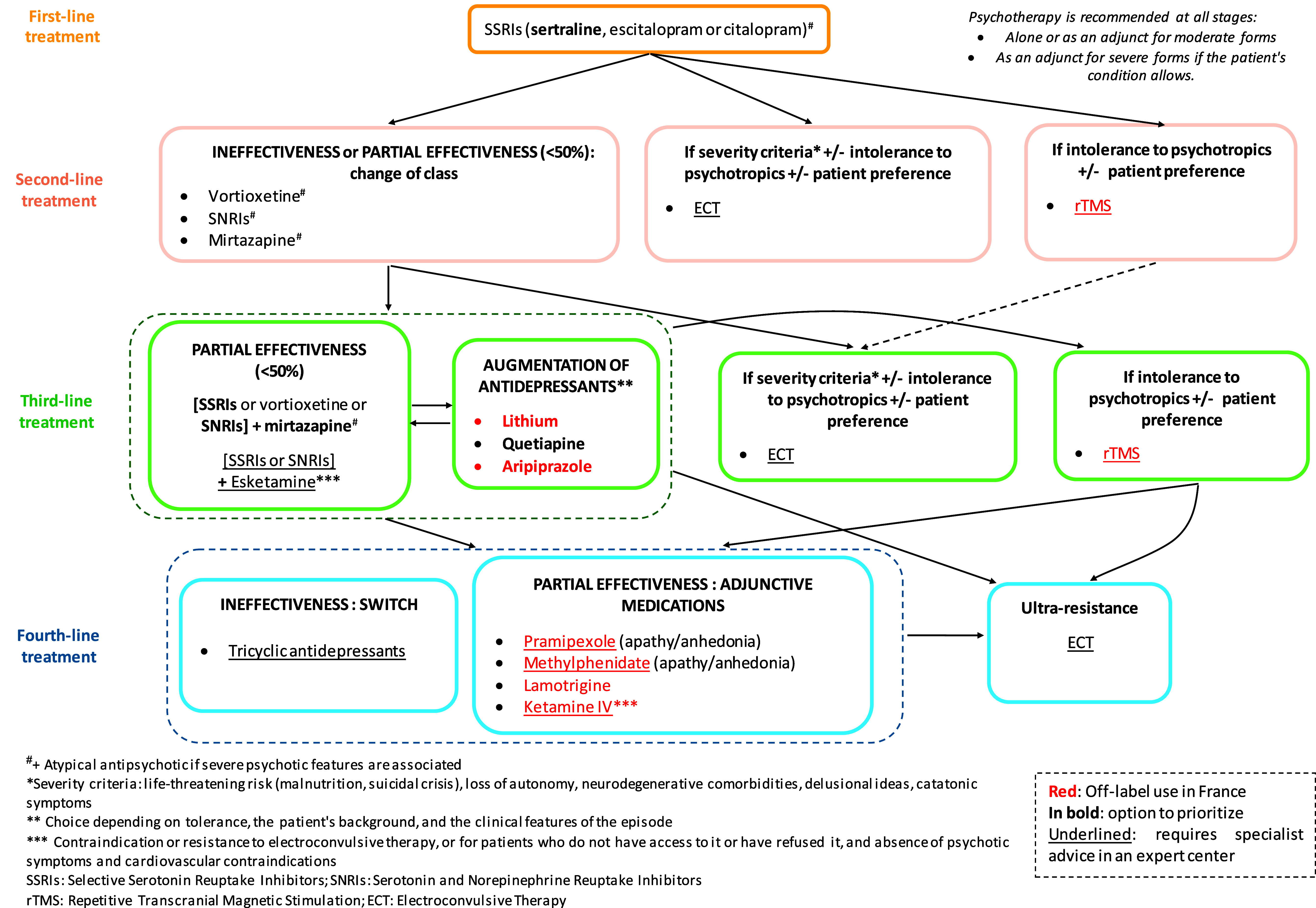
Decision tree for the therapeutic management of moderate to severe major depressive episodes in the older adults. Figure 2. long description.

The final tool represents a comprehensive synthesis of expert opinion, informed by current evidence and clinical experience. It addresses not only medication selection but also crucial aspects such as dosing considerations, monitoring requirements, management of comorbidities, and treatment-resistant cases. Table 2 presents the complete set of 57 validated items organized into 10 clinically relevant sections. The decision tree (Figure 2) provides a stepped-care approach for managing moderate to severe major depressive episodes in older adults. The algorithm prioritizes treatments with the most favorable benefit–risk ratio for this population. Sertraline, citalopram, and escitalopram are recommended as first-line treatments due to their favorable safety profile, lower risk of drug–drug interactions, and available information from clinical trials. The algorithm then progresses through various augmentation strategies or medication switches based on response, with specific guidance on monitoring and safety considerations at each step.

**Table 2.** Final tool Table 2. long description.

Prescription framework for antidepressants	
1	Before prescribing an antidepressant in an older adult, I look for cardiovascular comorbidities: arrhythmias or prolonged QT (reference ECG), blood pressure disorders: hypertension, hypotension, or orthostatic hypotension (related to standing).
2	Before prescribing an antidepressant in an older adult, I look for signs suggestive of a neurocognitive disorder (cognitive complaints from the patient or their surroundings, such as memory loss, word-finding difficulties, progressively worsening loss of autonomy).
3	Before prescribing an antidepressant in an older adult, I review the medications already prescribed to the patient, particularly: medications with a high anticholinergic load, with a sedative effect, with QT-prolongation potential, anticoagulants or antiplatelet agents, tramadol, strong enzyme inducers or inhibitors (HUG table [54]).
4	Before prescribing an antidepressant, I check for medications that could cause depressive symptoms (corticosteroids, antiretrovirals, antimalarials, hormonal treatments, or immunotherapy), trying to find an alternative or attempt a dose reduction.
5	Before prescribing an antidepressant, I search for and eliminate an organic cause of depression (OSAS, hypothyroidism, hypercortisolism, neurological diseases, autoimmune, inflammatory, addiction, etc.)
6	Before prescribing an antidepressant in an older adult, I look for other comorbidities of interest: hyponatremia (<130 mmol/L); narrow-angle glaucoma; urinary retention; recent bleeding history (<1 month).
7	Before prescribing an antidepressant in an older adult, I check for the presence of renal insufficiency (CrCl <60 mL/min) or liver diseases causing hepatic insufficiency (e.g., cirrhosis or hepatitis).
8	Before prescribing an antidepressant in an older adult, I evaluate the risk of falls: falls within the last 6 months, walking functionality.

I want to prescribe an antidepressant to an elderly person. How do I do it?	
9	I do not prescribe tricyclic antidepressants (TCA) as first-line treatment.
10	For older patients, based on international evidence levels, efficacy, and tolerance, I preferentially recommend mirtazapine over mianserin (National Drug Safety Agency alert on the risks of sedation, agranulocytosis, hepatitis, and seizures).
11	In the case of a major depressive episode (MDE), I prefer to prescribe an SSRI as first-line treatment, particularly sertraline, which has the best benefit/risk ratio.
12	In the case of MDE, I can also prescribe mirtazapine as first-line, particularly when an orexigenic effect is desired, or rapid action on insomnia is needed if hygiene and dietary rules have been insufficient.
13	In the case of failure of an SSRI or mirtazapine, and in the absence of poorly controlled hypertension in the patient (contraindication for SNRIs), I prescribe an SNRI, paying particular attention to the patient’s initial level of anxiety.
14	I avoid prescribing antidepressants with a high anticholinergic load (TCA, antidepressants, paroxetine).
15	In the case of concomitant or recent (<1 month) hyponatremia (<130 mmol/L), I review the use of thiazide diuretics, ACE inhibitors, or laxatives, which are also known to cause hyponatremia.
16	In the case of hyponatremia (<130 mmol/L), before prescribing or changing medication, I search for the cause and correct it. If the cause is linked to an SSRI or SNRI, mirtazapine may be an alternative as it carries a lower risk of induced hyponatremia.
17	I start antidepressants gradually, in increments of 25 to 50% of the target dose every one to two weeks, depending on available forms, episode severity, and tolerance. I reevaluate the dose every 4 to 12 weeks.
18	Antidepressants can (or must, in the case of partial response) reach the maximum recommended dose in adults. Dose adjustments will differ in the following cases: age > 85 years, impaired renal/hepatic function, comorbidities, and based on tolerance (absence of hyponatremia under SSRIs, no falls, confusion, or digestive disturbances). *(Maximum doses according to the SPC: sertraline 200 mg/day, escitalopram 10 mg/day, citalopram 20 mg/day, fluvoxamine 300 mg/day, duloxetine 120 mg/day, venlafaxine 300 mg/day, milnacipran 100 mg/day, mirtazapine 45 mg/day, mianserin 90 mg/day, vortioxetine 20 mg/day).*
19	Every 1 to 3 months until stabilization, then every 3 to 6 months, I perform clinical monitoring for tolerance: confusion, falls, and other adverse effects.
20	Every 1 to 3 months until stabilization, then every 3 to 6 months, I perform clinical monitoring for efficacy (searching for symptom improvement).
21	For monitoring, I perform a complete blood count if prescribing mianserin when the patient presents with fever, sore throat, or other signs of infection.
22	After complete or partial clinical improvement of depression, and in the presence of cognitive complaints from the patient or their surroundings, I perform a screening cognitive test (e.g., MMSE, FAB (or BREF in French), or MOCA) and may request a full neuropsychological assessment at a memory center.
23	I ensure treatment adherence by asking the patient or their caregivers. In case of doubt and lack of treatment efficacy after more than 6 weeks of initiation, I can perform plasma drug level testing.
24	For the same episode*, I maintain treatment for at least 1 year after achieving the effective dose if it is the first depressive episode, 2 years if it is the second, and at least 3 years or even lifelong if it is the third or more recurrent episode. *(Two episodes are considered different if they are separated by at least 6 months without symptoms)
25	I prescribe a complete stop of the treatment according to a tapering protocol of 25% of the initial dose in increments of 2 to 5 weeks, depending on the medication (higher risk of discontinuation syndrome if half-life is less than 24 hours, such as venlafaxine, duloxetine, or paroxetine) and the risk of discontinuation syndrome* in the patient. *Risk factors for discontinuation syndrome: previous discontinuation syndrome due to non-adherence, failure of previous attempts to stop, use of medications at doses higher than prescribed, long duration or high dose of treatment.

In case of cardiovascular pathologies	
26	In case of hypertension, I prefer an SSRI (particularly sertraline) or mirtazapine.
27	In case of hypotension, I prefer an SSRI (particularly sertraline) and implement general recommendations for managing hypotension (compression stockings, check for dehydration or malnutrition…).
28	In case of controlled hypertension, I am cautious about increasing the doses of SNRIs (especially venlafaxine beyond 150 mg/day), and I monitor blood pressure at each dosage increase.
29	In case of heart disease, I prefer first-line treatment with an SSRI like sertraline or mirtazapine and avoid TCA. The use of SNRIs is possible in the second line with caution.
30	In patients with arrhythmia, prolonged QT, or hypokalemia, I do not prescribe TCA, escitalopram, or citalopram.
31	After initiating a medication, I perform an ECG check after 1 week in patients with arrhythmia or in cases of borderline or prolonged QT.
32	When prescribing an antidepressant, I check for other medications that might cause episodes of hypotension (alpha1-blockers, anxiolytics, etc.).

In case of altered neurocognitive assessment	
33	If a patient has been on a long-term prescription of an antidepressant with a high anticholinergic load and presents with early confusion or early onset of MCI, I prefer switching to an antidepressant with a lower anticholinergic load

In case of renal insufficiency	
34	In moderate renal insufficiency (30 < CrCl<60 mL/min), I start paroxetine at half the recommended initial dose (10 mg/day) and increase slowly according to tolerance. I do not prescribe milnacipran at doses >50 mg/day.
35	In severe renal insufficiency (15 < CrCl<30 mL/min) or terminal renal insufficiency (CrCl<15 mL/min) for SNRIs: - I do not prescribe duloxetine. - The maximum dose of venlafaxine is 187.5 mg/day. - The maximum dose of milnacipran is 25 mg/day. If dose escalation is necessary (e.g., partial efficacy), plasma drug level monitoring is appropriate.
36	In severe renal insufficiency (15 < CrCl<30 mL/min) or terminal renal insufficiency (CrCl<15 mL/min), among SSRIs, I prefer sertraline, escitalopram, and citalopram, starting treatment at a minimal dose and increasing very gradually based on clinical tolerance. If fluoxetine, fluvoxamine, or paroxetine is already being used, it may be maintained at the same dosage with clinical tolerance monitoring. If dose escalation is necessary (e.g., partial efficacy), plasma drug level monitoring may be considered if available locally.
37	In case of renal insufficiency, I start mirtazapine at 15 mg and increase it very gradually according to tolerance.

In case of hepatic insufficiency	
38	In case of hepatic insufficiency or if transaminases are more than 3 times the upper limit of normal, or if prothrombin time is <50%, I do not prescribe agomelatine, duloxetine, or mianserin.
39	In case of hepatic insufficiency, I prefer antidepressants with low hepatic extraction rates, such as fluoxetine or citalopram at reduced doses (half the usual dose) or milnacipran without dose adjustment.

In case of co-prescriptions of interest	
40	I avoid adding an antidepressant with a high anticholinergic load (TCA, paroxetine) in cases of a significant overall prescription load (score > 5 on the AIS scale modified by Javelot et al. in 2022). If the anticholinergic load of the prescription is already high, I look for alternatives to reduce it. For antidepressants, prefer another SSRI or SNRI, or mirtazapine, or vortioxetine.
41	In the case of polypharmacy, I look for possible drug interactions by consulting the available tools: Vidal® Interactions ANSM Thesaurus HUG Table DDI Predictor® Drugs® REMEDI[e]S List (Roux et al. 2021 [13]) Lexi-comp® Prescription assistance software analysis tool
42	I avoid prescribing an SSRI, SNRI, or tricyclic antidepressant in the case of concomitant tramadol prescription. I reassess the indication for analgesic treatment. If continuation is necessary, I monitor and educate the patient on symptoms to watch for (diarrhea, tachycardia, sweating, tremors, confusion).
43	In the case of concomitant use of an anticoagulant or antiplatelet drug and antidepressants, I perform close monitoring of the usual anticoagulant treatment parameters to adjust the dosages appropriately.
44	In the case of prescribing an SSRI, SNRI, or vortioxetine, I ensure and educate the patient to avoid concomitant use of non-steroidal anti-inflammatory drugs, which also increase the risk of bleeding.
45	In the case of recent bleeding, particularly gastrointestinal, I prefer prescribing a weakly serotonergic antidepressant, mirtazapine as the first choice, SNRI as the second.

In case of other comorbidities of interest	
46	In the case of urinary retention or closed-angle glaucoma, I avoid prescribing an antidepressant with a high anticholinergic load and prefer an SSRI other than paroxetine, an SNRI, or mirtazapine.

In case of failure of first-line treatment (monotherapy)	
47	I can prescribe vortioxetine as the second or third-line treatment in older patients with depression.
48	If a first antidepressant at an effective dose has only resulted in a partial effect despite 9–12 weeks of proper treatment adherence, I switch antidepressants, favoring another class. I may also consider plasma drug level testing, if possible, once the target dose is reached, to rule out the possibility of suboptimal plasma dosing.

In case of characterized depressive episode with resistance	
49	I never prescribe 3 antidepressants in combination.
50	I do not prescribe 2 SSRIs or 2 SNRIs, or 1 SSRI +1 SNRI, or an SSRI or SNRI with an MAOI or a tricyclic antidepressant.
51	After failure of 2 properly conducted monotherapies or a partial response to an antidepressant at an effective dose, I may consider a second-line combination therapy of an SSRI or SNRI with mirtazapine. The adjustment of dosages will then be guided by clinical factors (tolerance and efficacy). The use of the recommended maximum dose for both antidepressants in adults is possible.

In case of failure or partial efficacy of antidepressant combination therapy	
In this context (failure or partial efficacy of antidepressant combination therapy), I request a specialist’s opinion.	
52	I can temporarily add, as a third-line treatment and in the absence of metabolic, neurological, or vascular contraindications, an antipsychotic to antidepressant monotherapy: first-line, aripiprazole at a low dose (titrating 1–2 mg per week in oral solution up to a maximum of 5–10 mg/day) or second-line, quetiapine 50–150 mg/day (lower tolerance and efficacy).
53	In case of failure after two lines of treatment or antidepressant combination therapy, I can add lithium salts as a third-line treatment, aiming for a residual lithium level of 0.4–0.6 mmol/L, in the absence of renal insufficiency (<60 mL/min unless authorized by the nephrologist based on the benefit–risk ratio)*. *Especially in cases of high recurrence of suicidal history, good familial response to lithium, or suspicion of bipolar spectrum (personal or familial).
54	I can propose lamotrigine (off-label) in cases of resistant depression in older patients, as a third or fourth-line treatment (in case of failure or contraindication to lithium or aripiprazole), particularly in cases of doubt about a potential attenuated bipolar disorder. I introduce it in weekly titrations of 25 mg up to an effective dose, monitoring for the appearance of cutaneous toxicity.
55	I can prescribe clomipramine as a fourth-line treatment at a low dose (starting at 10 mg with gradual increases up to a maximum of 75 mg/day) in the absence of neurocognitive or cardiovascular disorders, and preferably under hospital supervision.
56	In case of depression resistant to four lines of treatment (monotherapies and antidepressant combination therapy) or in cases of depression in an older patient with life-threatening risks (malnutrition, suicidal crisis, pressure ulcer complications), loss of autonomy, neurodegenerative comorbidity, delusional ideas, or catatonic symptoms, I refer my patient to an ECT service for consultation as soon as possible.
57	In case of depression resistant to two lines of treatment or in cases where it is impossible to change or increase psychotropic medications, I refer my patient to an rTMS service for consultation (in the absence of a suicidal crisis and psychotic symptoms).

ACE, angiotensin-converting enzyme inhibitor; AIS, Anticholinergic Impregnation Scale; ANSM, French national drug safety agency; CrCl, Creatinine clearance; DDI, drug–drug interaction; ECG, electrocardiogram; ECT, eclectroconvulsivotherapy; FAB, frontal assessment battery; HUG, Geneva University Hospitals; MAOI, monoamine oxidase inhibitor; MCI, Mild Cognitive Impairment; MMSE, Mini-Mental State Examination; MOCA, Montreal Cognitive Assessment; OSAS, Obstructive Sleep Apnea Syndrome; REMEDIES, Review of potentially inappropriate Medication prescribing in Seniors; rTMS, Repetitive Transcranial Magnetic Stimulation; SNRIs, Serotonin and Norepinephrine Reuptake Inhibitors; SPC, Summary of Product Characteristics; SSRIs, Selective Serotonin Reuptake Inhibitors.

## Discussion

This Delphi survey, conducted with experts in psychotropics, old age psychiatry, and geriatrics, produced the first French recommendations in this field. These recommendations constitute an initial step toward a comprehensive decision-support tool for antidepressant prescribing in older adults, specifically designed for primary care professionals. They provide a foundation for the co-design of this future tool, involving both general practitioners and patients, to facilitate shared decision-making, reduce the risk of drug-related iatrogenesis, and optimize the management of major depressive disorder in this vulnerable population.

### Expert consensus process and outcomes

More than 20 experts participated in each round, representing diverse specialties and a majority of experienced professionals (69.6% with over 10 years of experience). Their geographic distribution ensured a broad representation of national practices, leading to a robust consensus on the proposed items. The Delphi method is a well-established approach that has been successfully applied in mental health and other medical fields to obtain a consensus [24]. For example, a similar survey conducted by Christi et al which suggests clinical recommendations that could be useful in the treatment with lithium of older patients [60]. While no specific methodology is defined, the use of a Likert scale and the percentage of agreement between experts are commonly used. We chose a percentage agreement between the experts of 80%, which is part of the high threshold that can be found in Delphi studies. We were looking for a strong consensus between the experts. The number of rounds in this survey (four) aligns with what is typically expected in Delphi methodology. A panel of at least 20 participants, as achieved in our study, is generally recommended to ensure stable results in item scoring [24, 61, 62].

The final tool aligns with various international recommendations on this subject [3, 25–27, 36–39]. Establishing precise recommendations is challenging due to the limited and sometimes contradictory data from studies on antidepressants in older adults. As a result, some items sparked opposing views among participants, with consensus allowing for their validation. Developing specific items on initiation, dose escalation, or monitoring is also difficult, as no consensus exists in the literature. For example, the preference for sertraline as a first-line agent aligns with the systematic review by Kok and Reynolds, which noted its favorable efficacy and tolerability profile in older adults [3]. Interestingly, our expert panel’s preference for escitalopram contrasts somewhat with the placebo-controlled trials reviewed by Mulsant et al., which failed to demonstrate the superiority of escitalopram and citalopram to placebo in late-life depression [28]. This discrepancy highlights the importance of both evidence-based medicine and clinical expertise in developing practical guidelines, as expert opinion may sometimes precede or complement published evidence in guiding clinical practice. Treatment with mirtazapine appears to be a promising option when depression is associated with decreased appetite or sleep disturbances. It also represents a valuable second-line option following SSRI failure [27, 63].

The themes identified for classifying the items align with the common comorbidities found in older adults. Cardiovascular diseases, particularly hypertension, are common in this population. Public Health France estimates the prevalence of hypertension among individuals aged 65–74 years at over 60% [64]. Arrhythmias are also highly prevalent in this group. The side effects of antidepressants can exacerbate these pre-existing conditions, making proper adaptation essential [47, 48]. Mild cognitive disorders affect between 20 and 40% of those over 65 years old [64]. While liver insufficiency is rare, kidney insufficiency is more common, and antidepressant dosages should be adjusted according to the patient’s renal function to limit the risk of overdose, which could lead to side effects. The Esteban study (2014–2016) highlights a 6.5% prevalence of chronic kidney disease with a glomerular filtration rate below 60 mL/min in those aged 65 to 74 years [65].

It was crucial to emphasize the importance of checking for drug interactions, as older patients are often on multiple medications. In a 2018 study, 26.3–39.9% of people over 65 in Europe were taking more than five medications daily [66]. Pharmacokinetic interactions are possible, as molecules like paroxetine, fluoxetine, or duloxetine inhibit certain cytochrome P450 enzymes involved in the metabolism of other drugs [67]. Clinically significant interactions often involve additive effects: excessive sedation, anticholinergic effects, and serotonin syndrome [68].

Items that achieved validation despite a more moderate expert agreement (between 80% and 90%) consensus included those related to diagnostic tests (e.g., ECG) and functional assessments (e.g., walking test, fall risk assessment, cognitive tests), acknowledging the practical challenges in private practice. A lack of time during consultations is a current challenge faced by general practitioners in caring for their patients [69]. Thus, performing these tests or questionnaires may not always be feasible in primary care settings. The goal, therefore, is to raise awareness among prescribers about the importance of these measures and the associated risks.

Disagreements also arose regarding the choice of antidepressants in cases of prolonged QT interval, hypokalemia, or arrhythmias. Here, identifying the underlying cause, evaluating the benefit–risk ratio, and seeking cardiology advice are essential. Venlafaxine’s cardiotoxicity was debated, particularly regarding its potential to prolong the QT interval. The decision was made not to list this molecule among those to avoid in such cases but rather to prioritize duloxetine among SNRIs [59]. Risks are indeed higher with tricyclic antidepressants or (es)citalopram, and they seem to vary for venlafaxine across studies [46, 59].

The prescription of antidepressants in cases of renal insufficiency also sparked discussion, particularly regarding plasma drug concentration monitoring. Some experts do this routinely, while others do not, either because the technique is not readily available or because they consider that no therapeutic concentration ranges exist for older adults. The need for plasma drug monitoring after 9 weeks of treatment inefficacy was debated. A 12-week evaluation period, with a focus on adherence, appeared more appropriate.

Vortioxetine was initially proposed as a viable alternative in most cases during the first round. However, although it is considered beneficial in terms of cognitive effects [70], its position was reconsidered with the experts during the subsequent rounds due to the limited studies specifically in older patients. Indeed, it has a unique pharmacological profile with a so-called multimodal action [71]. Its efficacy seems similar, if not better, to that of SSRIs, as well as its tolerance, but the experience is insufficient for many participants, particularly in older adults [72, 73]. Therefore, its position was retained as a second-line option.

Given the numerous risks associated with clomipramine use in older adults, including anticholinergic side effects, increased risk of hypotension, and arrhythmias [16], and its absence from many guidelines, such as those from NICE [74] and CANMAT [39], it should likely be reserved as a last-line option. Its administration should only occur after specialized consultation and under close supervision, ideally in a hospital setting. The same applies to MAOIs.

### Management of treatment-resistant depression

The issue of pharmacoresistance was also addressed. The response rate to a monotherapy antidepressant trial is around 40% [75], and the frequency of major depressive episodes resistant to two well-conducted treatment lines (effective dose and sufficient duration) is around 30% [76]. In such cases, strategies are defined to increase the chances of remission [27, 32, 33, 36].

Our approach to treatment-resistant depression aligns with the algorithm proposed by Mulsant et al, which suggests a systematic progression through medication options with careful consideration of side effect profiles [28]. While our expert panel reached consensus on augmentation with atypical antipsychotics and lithium for treatment-resistant cases, there was insufficient consensus regarding newer approaches such as esketamine, ketamine, and methylphenidate combinations. This reflects the limited evidence specifically in geriatric populations and highlights the need for further research on these promising treatments for older adults with depression resistant to conventional approaches. The OPTIMUM trial results support our recommendations for aripiprazole augmentation, showing it to be significantly more effective than switching to bupropion in the short-term, with remission rates of 28.9% versus 19.3%, respectively [77]. This supports our stepped-care approach, recommending augmentation strategies in certain clinical scenarios. Interestingly, the OPTIMUM trial also found that lithium augmentation and switching to nortriptyline showed similar effectiveness in patients who had failed first-step treatments, consistent with our recommendations for these agents as later treatment options.

Electroconvulsive therapy (ECT) remains the gold standard for treating severe or treatment-resistant depression in older adults. Psychotic symptoms, treatment resistance or intolerance, catatonic features, acute suicidal ideations, and rapidly deteriorated physical status are well-established indications for this treatment. Repetitive Transcranial Magnetic Stimulation (rTMS) is appropriate following the failure of antidepressant treatment in cases of patient preference or intolerance to psychotropic drugs and should probably be considered prior to pursuing ECT. Indeed, patients who do not respond to electroconvulsive therapy (ECT) often show limited or no response to repetitive transcranial magnetic stimulation (rTMS) [78].

### Safety considerations in antidepressant selection

Safety concerns are important when prescribing antidepressants to older adults, as they represent a category with higher iatrogenic risks with advancing age. As highlighted by Kok and Reynolds, common risks include falls, hyponatremia, gastrointestinal bleeding, and cardiovascular effects [3]. The complexity of late-life depression management requires clinicians to balance efficacy with safety concerns while considering the multiple comorbidities and concomitant medications common in this population. Our tool specifically addresses monitoring for antidepressant adverse events, with, for example, recommendations for regular sodium level monitoring with SSRIs (items 15–16). Items 19–23 address the importance of clinical monitoring for both efficacy and adverse effects every 1–3 months until stabilization, then every 3–6 months. This approach is supported by evidence showing that the frequency, duration, and quality of follow-up visits significantly impact treatment outcomes [3]. Indeed, their meta-analysis found that additional follow-up visits could account for 27% of the improvement observed with antidepressants, highlighting that medication efficacy cannot be separated from the process of care. The risk of falls is particularly addressed through recommendations for physical examination and cautious dosing (items 8 and 17).

Furthermore, Mulsant et al. emphasize that the efficacy of antidepressants depends largely on how they are used, with poorer outcomes observed under usual care conditions compared to a systematic approach guided by a treatment algorithm [28]. This drug class is often misused or even underutilized in the older population [79]. First-line treatments are not always optimal, dosages and treatment durations are rarely optimized to be fully effective, and the patient’s overall health status is sometimes overlooked, especially in those with polypharmacy. In a study by Boehlen et al., 77.3% of patients aged 55– 84 years with depressive symptoms were untreated, despite three-quarters having at least two chronic conditions. Only 52.9% of antidepressant prescriptions were at the optimal dose [80]. Items 17, 18, or 25 are examples of reminder items on the correct use of antidepressants in older adults.

Any discussion of safety considerations in this context must acknowledge the crucial role that clinical pharmacists can play in the care of older adults with depression. While this practice is still developing in France, European studies have demonstrated the positive impact of pharmaceutical interventions, specifically in managing drug interactions, reducing potentially inappropriate prescriptions, and improving patient adherence to treatment and necessary follow-up [5]. Furthermore, clinical pharmacists are essential for fostering interprofessional collaboration, serving as a vital link between primary care professionals, including home nurses, general practitioners, and community pharmacists.

### Items lacking consensus and future research directions

Among the items not validated in this tool, we can mention the monitoring of sodium levels at 15 days after initiating antidepressant treatment, then at 30 days or based on clinical red flags. The variable onset of the syndrome of inappropriate antidiuresis and the practical implementation of this monitoring were the main points of disagreement.

The evaluation of osteoporosis risk did not seem applicable in practice unless, according to the experts’ experience, day hospitals can assess it in cases of recent falls or high fall risk.

Lavretsky et al. showed promising effects of combining methylphenidate and escitalopram in older adults with treatment-resistant depression [81]. Consensus was not reached on the type of depression and comorbidities that would benefit from this treatment.

Items concerning the management of treatment-resistant depressive disorders and potential augmentation strategies did not achieve consensus. The use of esketamine and ketamine in this population remains a subject of ongoing debate. While these agents show promise in treating treatment-resistant depression, a frequent and potentially life-threatening condition, robust evidence is still limited [82]. The TRANSFORM-3 study, for instance, did not demonstrate significant results, maybe due to a suboptimal dosage (max. 56 mg/session) compared with younger adults [83]. However, a recent systematic review (based mostly on small-sized and open-label trials) reported preliminary findings of symptomatic improvement in this challenging condition, with rare adverse events or discontinuations due to side effects [84]. Given that esketamine has been authorized for hospital use in adults in France, it is important not to exclude patients a priori based solely on age, particularly in severe episodes where alternative treatment options are lacking. The current lack of consensus in this population reflects the limited availability of real-world safety data.

There is limited data on pramipexole in late-life depression, although meta-analyses suggest its efficacy in treatment-resistant depression in adults [85]. This discussion focused on the extended-release form of pramipexole, which not all experts agreed with. The maximum dose of 1.05 mg/day was also debated, especially in cases of comorbid Parkinson’s disease.

Agomelatine and tianeptine were not included based on open-ended questions from the first round. Agomelatine has a contraindication for those over 75 due to a lack of data in this age group and liver risks [86], while tianeptine carries a high risk of drug dependence, necessitating a specific prescribing framework [87].

### Limitations and implementation considerations

Although the Delphi method is a validated approach to obtaining expert consensus, there are points of improvement. This study was conducted among French-speaking experts, which limits the generalizability of the recommendations obtained. However, this consensus among French experts was essential in order to then be able to compare the tools used in other European countries. Regarding the development of items, different molecules were grouped in the same item and separating them by molecule might have been relevant to ensure validation for each drug in the various proposed contexts. As 91.3% of the experts were hospital-based, the tool’s applicability in primary care has yet to be fully evaluated. Although few general practitioners participated, the study aimed to reach a consensus among experts in late-life depression to address issues frequently encountered by GPs. Future research should assess the tool’s acceptability and feasibility in routine primary care. It might have been useful to specify the strategy more clearly in cases of treatment ineffectiveness or partial effectiveness, particularly with regard to increasing doses or switching molecules.

## Conclusion

The development of this tool serves as the foundation for expert-driven guidelines on the safe and effective use of antidepressants in older adults, established by the SF3PA, SFPC, and SFGG. By providing precise and pragmatic recommendations tailored for real-world clinical practice, it addresses the complexities of prescribing in late-life depression, complementing existing decision-support tools. These guidelines aim to optimize antidepressant treatment, minimize drug-related iatrogenesis, and enhance patient outcomes. Further evaluation is needed to assess its real-world impact on improving antidepressant efficacy and patient safety in geriatric populations, but its foundation in expert consensus and clinical guidelines underscores its potential as a valuable resource for healthcare providers.

## Data Availability

The datasets generated and/or analyzed during the current study are available from the corresponding author on reasonable request.

## Long descriptions

### Table 1. Long description

The table consists of five columns: the first lists characteristics, followed by columns for the 1st round (n = 23), 2nd round (n = 20), 3rd round (n = 21), and 4th round (n = 21). Data is presented as n (%).

* Specialty: Geriatric psychiatrists represent the majority, increasing from 52.2% in round 1 to 61.9% in round 4. Hospital pharmacists remain stable around 24-26%. Geriatricians range from 9.5% to 15.0%. General practitioners decrease from 8.7% to 0% by round 4.

* Years of experience: Most participants have > 10 years of experience (61.9% to 75.0%). Those with < 5 years range from 19.0% to 28.6%, while the 5-10 year group is the smallest.

* Type of practice: Hospital-based practice is dominant, reaching 100% by round 4. Primary care practice starts at 8.7% and drops to 0%.

* Geographic distribution: Participants are primarily from French regions, with the highest concentrations in Ile-de-France (Paris), Occitanie, and Pays de la Loire. International representation includes single participants from Belgium, Monaco, and Switzerland.

Navigate back to Table 1..

### Figure 1. Long description

The flowchart consists of five main stages connected by downward-pointing arrows.

1. At the top, the first box is labeled 1st round: 53 proposed items. An arrow points down to the next stage, with text to the right indicating 20 validated items, 2 removed items, 31 modified items, and 15 added items.

2. The second box is labeled 2nd round: 46 proposed items. An arrow points down to the next stage, with text to the right indicating 19 validated items, 3 removed items, 24 modified items, and 2 added items.

3. The third box is labeled 3rd round: 26 proposed items. An arrow points down to the next stage, with text to the right indicating 13 validated items, 5 removed items, and 8 modified items.

4. The fourth box is labeled 4th round: 8 proposed items. An arrow points down to the final stage, with text to the right indicating 6 validated items and 2 removed items.

5. The final box at the bottom is double-outlined and labeled Final tool with 58 items including therapeutic decision tree.

Navigate back to Figure 1..

### Table 2. Long description

The table is divided into several thematic sections.

Prescription framework for antidepressants (Points 1 to 8): Focuses on pre-prescription assessments including cardiovascular comorbidities (arrhythmias, Q T prolongation, blood pressure), neurocognitive signs, medication review (anticholinergics, anticoagulants), organic causes of depression (hypothyroidism, O S A S), and fall risk.

Prescribing to an elderly person (Points 9 to 25): Recommends S S R I s like sertraline or mirtazapine as first-line treatments. It advises against tricyclic antidepressants (T C A) and details titration (25 to 50 percent increments), monitoring for efficacy and tolerance every 1 to 3 months, and maintenance durations (1 to 3 years depending on episode recurrence).

Cardiovascular pathologies (Points 26 to 32): Prefers sertraline or mirtazapine for hypertension/hypotension. Contraindicates T C A, escitalopram, or citalopram for patients with prolonged Q T or arrhythmias.

Neurocognitive assessment (Point 33): Advises switching to low anticholinergic load antidepressants if cognitive impairment is present.

Renal and Hepatic insufficiency (Points 34 to 39): Provides specific dose adjustments based on Creatinine Clearance (C r C l). For severe renal insufficiency, duloxetine is avoided while sertraline or citalopram are preferred at minimal doses. For hepatic insufficiency, agomelatine and duloxetine are avoided.

Co-prescriptions and Drug Interactions (Points 40 to 45): Lists tools for checking interactions (Vidal, A N S M, H U G) and warns against combining antidepressants with tramadol or N S A I D s due to bleeding risks.

Other comorbidities (Point 46): Addresses urinary retention and glaucoma.

Treatment Failure and Resistance (Points 47 to 57): Outlines second-line (vortioxetine, S N R I), third-line (aripiprazole, lithium, or lamotrigine), and fourth-line (clomipramine) options. It concludes with referrals for E C T or r T M S in cases of severe resistance or life-threatening risks.

Navigate back to Table 2..

### Figure 2. Long description

The flowchart is organized into four horizontal rows representing treatment stages.

Top row: First-line treatment. A central box indicates S S R I s (sertraline, escitalopram or citalopram). A note at the top right specifies that psychotherapy is recommended at all stages, either alone or as an adjunct.

Second row: Second-line treatment. Three boxes branch from the first-line. Left: Ineffectiveness or partial effectiveness (<50%) leads to a change of class to Vortioxetine, S N R I s, or Mirtazapine. Middle: If severity criteria or intolerance to psychotropics exist, E C T is recommended. Right: If intolerance to psychotropics exists, r T M S is recommended.

Third row: Third-line treatment. Four boxes. Left: Partial effectiveness (<50%) leads to combining [S S R I s or vortioxetine or S N R I s] with mirtazapine, or [S S R I s or S N R I s] with Esketamine. Middle-left: Augmentation of antidepressants with Lithium, Quetiapine, or Aripiprazole. Middle-right: E C T for severity or intolerance. Right: r T M S for intolerance.

Fourth row: Fourth-line treatment. Three boxes. Left: Ineffectiveness leads to a switch to Tricyclic antidepressants. Middle: Partial effectiveness leads to adjunctive medications including Pramipexole, Methylphenidate, Lamotrigine, or Ketamine I V. Right: Ultra-resistance leads to E C T.

Footnotes define severity criteria as life-threatening risk, loss of autonomy, or neurodegenerative comorbidities. Initialisms include S S R I s for Selective Serotonin Reuptake Inhibitors, S N R I s for Serotonin and Norepinephrine Reuptake Inhibitors, r T M S for Repetitive Transcranial Magnetic Stimulation, and E C T for Electroconvulsive Therapy.

Navigate back to Figure 2..
